# Pneumatic versus liquid enema reduction in the management of pediatric intussusception: an updated systematic review and meta-analysis

**DOI:** 10.1097/MS9.0000000000003754

**Published:** 2025-09-04

**Authors:** Ruaa Mustafa Qafesha, Israa Sharabati, Muataz Kashbour, Menna Elbadry, Hatem Eldeeb, Baraa M. Ayesh, Asmaa Elganady, Mahmoud Shaaban Abdelgalil, Mohammed Abdulrazzak, Afnan W. M. Jobran

**Affiliations:** a Faculty of Medicine, Al-Quds University, Jerusalem, Palestine; b Medical Research Group of Egypt, Negida Academy, Arlington, Massachusetts; c Diagnostic Radiology Department, National Cancer Institute, Misrata, Libya; d Faculty of Medicine, October 6 University, 6th of October City, Egypt; e Faculty of Medicine, Al-Azhar University, Cairo, Egypt; f Faculty of Medicine, Alexandria University, Alexandria, Egypt; g Faculty of Medicine, Ain-Shams University, Cairo, Egypt; h Faculty of Medicine, University of Aleppo, Aleppo, Syria

**Keywords:** enema reduction, intussusception, liquid reduction, meta-analysis, pediatric intussusception, pneumatic reduction

## Abstract

**Introduction and importance:**

Intussusception is the most frequent cause of bowel obstruction in infants. Most cases are managed nonsurgically with liquid or enema reduction. Our study aims to evaluate the efficacy and safety of air versus liquid enema reduction for the treatment of intussusception in pediatric patients.

**Materials and methods:**

A literature search was conducted on electronic databases, and the references of all included studies were searched to identify additional relevant studies meeting our inclusion criteria. Our meta-analysis included studies that compared the use of air and liquid enemas in pediatric patients with intussusception. The primary outcomes assessed were reduction success, perforation, and recurrence rates. Secondary outcomes included hospital stay duration, reduction time, reduction pressure, and fluoroscopy time. Meta-analysis was performed using RevMan 5.4.

**Results:**

Twenty-nine studies were included in the analysis, encompassing 9281 patients. The pneumatic reduction was significantly associated with a higher success rate (risk ratio: 1.22, 95% confidence interval [CI]: {1.12, 1.34}) and a shorter reduction time (mean difference [MD]: −2.64, 95% CI: [−4.73, −0.55]). No significant difference was found between the two procedures in terms of perforation rate, recurrence rate, length of hospital stays, reduction pressure, and fluoroscopy. Subgroup analysis revealed that pneumatic reduction had higher success rates in both RCTs and cohort studies and was superior to liquid reduction before the year 2011. After 2011, both techniques had similar success rates. Pneumatic reduction also showed higher success rates in both children’s and non-children’s hospitals and among the general radiologists’ subgroup, though pediatric radiologists achieved similar success rates with both techniques.

**Conclusions:**

Pneumatic enema reduction offers higher success and faster treatment for pediatric intussusception, with comparable safety to hydrostatic methods, supporting its use as the preferred nonsurgical approach. Further comparative studies are recommended to examine radiation dose and fluoroscopy time.

## Introduction

Intussusception is a common and potentially life-threatening cause of intestinal obstruction in young children, occurring when a proximal segment of the bowel telescopes into a distal segment. This condition primarily affects infants and toddlers, with the highest incidence observed between 3 and 24 months of age, followed by a gradual decline thereafter[1,2]. While most pediatric cases are idiopathic, a pathological lead point – such as Meckel’s diverticulum, polyps, or lymphoma – is identified in approximately 25% of cases[3]. At many institutions, ultrasonography is the preferred method for detecting intussusception because of its high sensitivity and specificity^[4,5]^. The approach to managing intussusception has undergone significant evolution in recent decades, transitioning from immediate surgical intervention to regular utilization of radiological reduction, resulting in reduced complications for patients. In the absence of contraindications, such as perforation, peritonitis, or hypovolemic shock, liquid (saline, contrast, and barium) or pneumatic (air) enemas are most commonly used under fluoroscopic or ultrasound guidance[6]. Intussusception can be reduced using either technique. The pneumatic involves using air pressure to push the telescoped segment of the intestine back into its normal position and is a very efficient option that offers extra benefits such as reduced cost and a lower perforation risk. Ultrasound-guided hydrostatic reduction offers an alternative approach that eliminates the necessity of exposing children to substantial radiation doses, unlike X-ray-monitored pneumatic reduction and barium reduction techniques[7].

The selection of enema type at various institutions may be influenced by the proficiency and confidence of practitioners, as well as the accessibility of resources. There is still ongoing debate regarding the optimal approach to decrease mortality and morbidity while maximizing success rates. The average intussusception hospitalization rate for children under 2 years of age is 112.9 per 100 000[8]. Thus, understanding the optimal enema reduction technique has implications for patient outcomes, medical education, and resource allocation.

Two prior meta-analyses have examined the topic of enema reduction for pediatric intussusception. The first, conducted in 2013[9], focused on only two outcomes, while the second, published in 2015[10], included single-arm studies, limiting the scope of their conclusions.

Given the increasing volume of comparative studies published in recent years, there is a need for a more rigorous and up-to-date synthesis of evidence. Therefore, the objective of this systematic review and meta-analysis is to comprehensively compare the efficacy and safety of pneumatic versus liquid enema reduction in the treatment of pediatric intussusception, evaluating multiple clinical outcomes across a wide range of study settings.

## Methods

We conducted this systematic review and meta-analysis after registration on PROSPERO. We strictly adhered to the Preferred Reporting Items for Systematic Reviews and Meta-Analysis (PRISMA 2020 version) guidelines and Cochrane Handbook for Systematic Reviews of Interventions during the preparation of this study^[11,12]^.

### Eligibility criteria

The studies included in this systematic review and meta-analysis satisfied the following PICOS criteria:

- Population: Pediatric patients who were under 18 years old and who underwent nonoperative reduction for intussusception.
- Intervention: Pneumatic enema reduction.
- Comparator: Liquid enema reduction.
- Outcomes: The primary outcome of this study is to compare the two procedures in terms of success rate. Secondary outcomes are recurrence, reduction time, perforation rate, length of hospital stay (LOHS), complications, and reduction pressure.
- Study design: We included randomized controlled trials (RCTs), nonrandomized clinical trials, and observational studies that compared the two procedures. Only English studies were included.

Studies were excluded if they did not meet the predefined inclusion criteria or were reported in any language other than English. We excluded book chapters, editorials, letters, conference abstracts, case reports, reviews, and single-arm studies.

**HIGHLIGHTS**

- This meta-analysis compares air versus liquid enema reduction for pediatric intussusception.
- Air enemas showed significantly higher success rates and shorter reduction times.
- No significant differences were found in safety outcomes like perforation or recurrence.
- Findings support pneumatic reduction as an effective and safe first-line treatment.

### Literature search and selection process

The PubMed, Web of Science, Cochrane Central Register of Controlled Trials, and Scopus databases were comprehensively searched from the inception of the study to November 2023 to identify eligible studies comparing pneumatic reduction to enema reduction in patients with intussusception. Furthermore, the reference lists of potentially eligible studies were manually screened to avoid missing potentially eligible studies.

The search query involved a combination of relevant keywords and their synonyms, including (“Intussusception” OR “Intestinal Invagination”) AND (“Air enema” OR “Pneumatic enema” Hydrostatic enema” OR “Barium enema” OR “Contrast enema” OR “Saline enema.” The search strategy was adapted to each database, and no restrictions or filters were applied. The full search strategy can be viewed in Supplemental Digital Content Appendix, available at: http://links.lww.com/MS9/A921.

Using Rayyan software (Qatar Computing Research Institute, 2016, Doha, Qatar)[13], six independent reviewers screened the titles and abstracts of the retrieved studies to assess their eligibility using the predefined inclusion criteria. Full-text articles of the potentially eligible citations were then assessed for final inclusion. The results were thoroughly revised, and any disagreements were resolved by a senior author.

### Data collection and management

Two authors performed independent data extraction using standardized Google Sheets (Alphabet, Inc., Google, USA). Subsequently, a senior author reviewed the sheets to identify any inconsistencies or discrepancies in the extracted data. The extracted data comprised separate items, including study characteristics (such as study ID, study location, study design, study duration, type of intervention, sample size, inclusion criteria, duration of symptoms, and main findings), baseline data (such as study group, age, gender, and duration of symptoms), and outcome measures. Specifically, the outcome measures evaluated were the success rate of the procedure, recurrence, reduction time, perforation rate, LOHS, mortality, reduction pressure, and fluoroscopy time.

### Risk of bias assessment/publication bias

For randomized trials, we assessed the risk of bias using the Cochrane risk-of-bias tool for randomized trials (ROB2)[14]. This tool is used to evaluate six domains for determining the risk of bias, and each domain evaluated as low risk, some concern, or high risk of bias. These domains are the randomization process, deviation from intended interventions, missing outcome data, measurement of the outcome, selection of the reported result, and other biases. For observation or cohort studies, the Newcastle‒Ottawa scale was used to assess the risk of bias[15]. Based on the effectiveness of the study group selection, comparability, and outcome assessment, this tool rates each study up to nine stars. Studies with seven or more stars were considered to have a low risk of bias, studies with four to six stars were considered to have a moderate risk of bias, and studies with fewer than four stars were considered to have a high risk of bias. Two authors separately rated the quality, and any debates were resolved by discussion and consensus.

### Primary and secondary endpoints

The primary endpoints included the overall success rate, perforation rate, and overall recurrence rate. The secondary endpoints comprised the LOHS (in hours), reduction time (in minutes), reduction pressure (in mmHg), fluoroscopy time, and mortality (number of deaths).

### Data synthesis and assessment of heterogeneity

To conduct the analysis of this study, RevMan software version 5.4.1 was used. For the dichotomous data, we used the pooled risk ratio (RR). For continuous data, we used a mean difference (MD) with a 95% confidence interval (CI). A *P* value <0.05 was considered significant. For heterogeneity, we used the *I*^2^ test and *P*-value. For the analysis to be considered heterogeneous, the *P*-value must be <0.1 or *I*^2^ >60%. To address any heterogeneity, we adopted a leave-one-out test or subgrouping.

A meta-regression analysis was conducted to investigate the influence of mean age and mean time of presentation on the success rate outcome. These meta-regression analyses were preceded by a standard pair-wise meta-analysis in which the pooled indexes of outcomes were estimated using OpenMeta [Analyst] software.

## Results

### Literature search results and characteristics of the included studies

**Figure 1.**
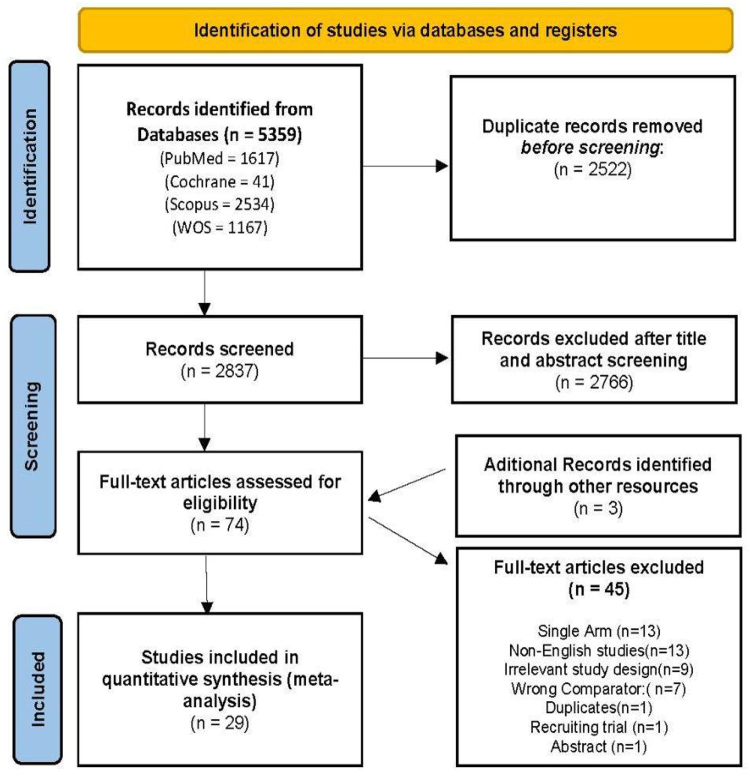
PRISMA flow diagram.

The search conducted across databases resulted in 5359 citations. After we removed the duplicates, 2837 were eligible for title and abstract screening. Twenty-six articles were finally included based on the eligibility criteria, after excluding 45 in the full-text screening phase. The references of the included studies were manually screened, and three additional articles were added. The detailed selection procedure can be found in the PRISMA flow diagram (Fig. 1).

Twenty-nine studies were included in our meta-analysis, for a total of 9281 procedures. Of these, 3932 (42.37%) of them were performed using the pneumatic arm, while the remaining 5349 procedures were performed using the hydrostatic arm. Six studies were RCTs, one was a prospective cohort study, and the remaining were retrospective. The used hydrostatic agents were either barium or saline, with a few studies using other liquids. Table 1 shows a summary of the included studies.

**Table 1:** Summary of the studies included in this systematic review and meta-analysis

Study ID	Country	Hospital Type	Study Design	Inclusion criteria	Enema Type	Sample size of each group		Performer		Guided By		Criteria used for technical success	Study duration
						*Liquid—Pneumatic*		*Liquid—Pneumatic*		*Liquid—Pneumatic*			
**Sun 2023**[16]	China	Non-Children	Retrospective	Less than 12 years from hospital database who were diagnosed with intussusception and managed with enema reduction	Cleansing Enema	780	113	Cleansing enema by a nurse, B-USGHE sonographer	Radiologist	Ultrasound	Fluoroscopy	Reflux of air into the terminal ileum and distal small bowel visualized directly by fluoroscopy fluid passed the cecum and been distributed in the small intestine, and no intussusception was found again by B-ultrasound examination	Jan 2019—Dec 2020
					Saline								
					Air								
**Chukwu 2022**[17]	Nigeria	Children	RCT	Children 1 to 12 months of age and presentation with uncomplicated intussusception confirmed by abdominal ultrasonography	Saline	26	26	Radiologist	Radiologist	Ultrasound	Ultrasound		Dec 2018—August 2020
					Air								
**Taher 2022**[7]	Egypt	Non-Children	Prospective single-blinded randomized comparative study	3 months- 3 Years of age, hemodynamically stable children with no significant abdominal distention, no clinical or radiological signs of peritonitis and symptoms less than 24 hours.	Saline	10	10			Ultrasound	Fluoroscopy		November 2020 to November 2021.
					Air								
**Miguel 2022**[18]	Spain	NA	Retrospective	Patients with ileocolic intussusception episodes diagnosed by US	Barium	380	98	Pediatric radiologists in the presence of pediatric surgeons		Barium: Fluoroscopy, Saline: Ultrasound	Fluoroscopy	Sonographic signs of successful reduction include the disappearance of the intussusception and the appearance of water and bubbles in the terminal ileum.	2005-2019
					Saline								
					Air								
**Chutiwongthanaphat 2021**[19]	Thailand	Children	Retrospective cohort	The diagnosis of intussusception by ultrasonography, having no contraindications, such as peritonitis or pneumoperitoneum, before a radiologist did enema reduction.	Saline	18	31	Radiologist	Radiologist	US	Fluoroscopy		Oct 2017—Feb 2019
					Air								
**Alehossein 2011**[20]	Iran	Children	Retrospective	Referred children to the radiology department for the treatment of intussusception which was proved by sonography	Barium	40	17	Radiologist	Radiologist	Barium: fluoroscopy	Fluoroscopy	flow of fluid via the ileocecal valve to the terminal ileum	1997- 2007
					Saline								
					Air								
										Saline: sonography			
**Yang 2021**[21]	China	Non-Children	Retrospective analysis	children aged 0–14 years; had one or more symptoms of intussusception and stable ultrasound indicated “concentric circle sign” and “sleeve sign”; no abdominal distension.	Saline	119	245			Ultrasound	Fluoroscopy	When water flowed through the ileocecum into the intestine, it showed a “crab claw sign” and a “honeycomb sign,” or When the intestine was developed and filled with air	Jan 2016 to May 2019
**Liu 2021**[22]	China	Children	Prospective Cohort	intussusception is diagnosed by ultrasound with characteristic image the onset time is less than 48 h aged between 4 months and 14 years a well general condition and no signs of peritonitis no clinical manifestations of small intestinal obstruction recurrence intussusception those who were diagnosed by intussusception again within 1 month	Saline	1119	1005	Pediatric surgeons	Radiologist	Ultrasound	Fluoroscopy	Disappearance of intussusception and visualization of air/normal saline from the cecum to the ileum through the ileocecal valve or an air/normal saline distended ileum.	2017-2018
					Air								
**Ali 2017**[23]	Egypt	Children	RCT	Hemodynamic stability. No marked abdominal distention. No clinical or radiological signs of peritonitis. Duration of symptoms less than 48 h. No clinical manifestations of small intestinal obstruction		40	40			Ultrasound	Fluoroscopy		Sep 2014—Sep 2015
**Xie 2017**[24]	China	Non-Children	RCT	Children 0-18 years who diagnosed with intussusception and visited the hospital emergency	Saline	62	62	pediatric surgeon	Radiologist pediatric surgeon	Ultrasound	Fluoroscopy	visualization of the normal saline or air from the cecum to the ileum through the ileocecal valve or normal saline or air-distended ileum and the disappearance of intussusception after reduction by ultrasound examination.	January 2014 to December 2015
					Air								
**Huai 2017**[25]	China	Non-Children	Retrospective	children with intussusception	Saline	75	107			Ultrasound	Ultrasound	Pneumatic: the entrance of a large amount of air into ileum and the disappearance of mass. hydrostatic = disappearance of “concentric circles” showed by ultrasound and the disappearance of resistance in the colon or ileocecal indicated the success of repositioning.	June 2011 to January 2016
					Air								
**Kaplan 2017**[26]	Germany	Non-Children	Retrospective	Patients with ileocolic intussusception episodes	Liquid contrast	17	30	Pediatric radiologist	Pediatric radiologist	Fluoroscopy	Fluoroscopy	Reduction of intussusception	2013-2015
					Air								
**Ntoulia 2016**[3]	USA	Children	Retrospective	all children who were referred to the radiology department at our institution for clinical suspicion of intussusception from 2005 to 2013	Isotonic water	224	270			200 Fluoroscopy + 24 US	Fluoroscopy		2005–2013
					Air								
**Khorana 2015**[27]	Thailand	Non-Children	Retrospective Cohort	Evidence of intestinal obstruction, intestinal invagination, and intestinal vascular compromise or venous congestion. abnormal nonspecific bowel gas pattern in the abdominal radiograph.	Barium	59	111	Radiologist	Pediatric surgeon, radiologist	Fluoroscopy	Ultrasound, Fluoroscopy	Disappearance of intussusception and the visualization of barium or air from cecum to ileum through ileocecal valve, or barium or air-distended ileum and absence of intussusception ultrasound examination.	2006-2012
					Air								
**Beres 2013**[9]	Canada	Children	Retrospective		Contrast	27	98				Fluoroscopy		Jan 2000—July 2010
					Air								
**Niramis 2010**[28]	Thailand	Children	Retrospective	All patients with intussusception treated over period of time even if the patients admitted for more than one episode of intussusception	Barium	349	697					Reduction of intussusception	1976-2008
					Air								
**Yalcin 2009**[29]	Turkey	Non-Children	Retrospective analysis	All patients diagnosed with intussusception	Barium	134	20	Radiologist	Radiologist			Reflux of barium or air into the terminal ileum.	1993- 2003
					Air								
**Kaiser 2007**[30]	USA	Children	Retrospective	Patients diagnosed by intussusception by either imaging (ultrasound, enema, computed tomography [CT]) or surgical exploration		65	125	Radiologist	Radiologist	Fluoroscopy	Fluoroscopy		1990-2007
**Rubi 2002**[31]	Spain	Non-Children	Retrospective	Every Child presented to the ER with suspicion of intussusception	Barium	130	21			Fluoroscopy	Fluoroscopy	Terminal ilium filled with air. The patient was given charcoal orally to look for it in the patient’s feces and confirm the result.	over a period of 21 years
					Air								
**Reid 2001**[32]	New Zealand	Non-Children	Retrospective	Children with intussusception admitted to the eight public hospitals in the South Island of New Zealand were identified from hospital record systems and departmental databases	Barium	34	42						June 1987 and December 1998
					Air								
**Hadidi 1999**[33]	Egypt	Children	RCT	All patients whose clinical and radiological data confirmed the diagnosis of intussusception were eligible for the study.	Barium	97	50	Pediatric Radiologist	Pediatric surgeon	Barium: Fluoroscopy Saline: US	Fluoroscopy	disappearance of intussusception and visualization of the passage of fluid and air bubbles from the caecum well into the terminal ileum.	July 1994—Dec 1997
					Saline								
					Air								
**Eshel 1997**[34](32)	Israel	Children	Retrospective		Barium	63	37						1986–1995
					Air								
**Ein 1997 [a]**[35]	Canada	NA	Retrospective	infant and child with an intussusception	Barium	336							1959–1968
**Ein 1997 [b]**[35]	Canada	NA	Retrospective	infant and child with an intussusception	Air		188						1985-1990
**Thomas 1993**[36]	China	Non-Children	Retrospective cohort	Intussusception patient		21	34			Fluoroscopy	Fluoroscopy		1988-1993
**Meyer 1993**[37]	USA	Children	RCT	All patients whose physicians had requested an enema examination except patient needed a special type of contrast agent	Barium	50	51	Pediatric radiologists		Fluoroscopy	Fluoroscopy	Successful reduction in less than 3 attempts	1989-1991
					Air								
**Palder 1991**[38]	Canada	Children	Retrospective	Children diagnosed with intussusception by clinical examination and history or plain radiograph over a period of 5 years	Barium	100	100			Fluoroscopy	Fluoroscopy	air or barium reflux some distance into the distal ileum	1986-1991
					Air								
**Beasley 1992**[39]	**Australia**	**Children**	Retrospective		Barium	200	187				Fluoroscopy		
					O2								
**Shiels 1991**[40]	USA	Children	Retrospective	pediatric patients who were first seen with clinically suspected intussusception.	Barium	100	75	Radiologist	Radiologist		Fluoroscopy	reflux of air into the small bowel and disappearance of the soft-tissue mass	1988-1990
					Water								
					Soulable Contrast								
					Air								
**Phelan 1988**[41]	Australia	Children	Retrospective	Episodes of intussusception	Barium	602	57				Fluoroscopy	Sudden flooding of the small bowel	Pneumatic: Jul1986—Mar1987 // liquid: 1970-1985
					O2								

IC ileocolic, IIC ileoileocolic, CC colocolic, II ileoileal

Two-thirds of the population was male (60%–70%). Most of the procedures are performed by radiologists under ultrasound or fluoroscopy. The baseline characteristics of the participants in each study are outlined in Supplemental Digital Content Table S1, available at: http://links.lww.com/MS9/A921.

### Quality assessment of included studies

**Figure 2.**
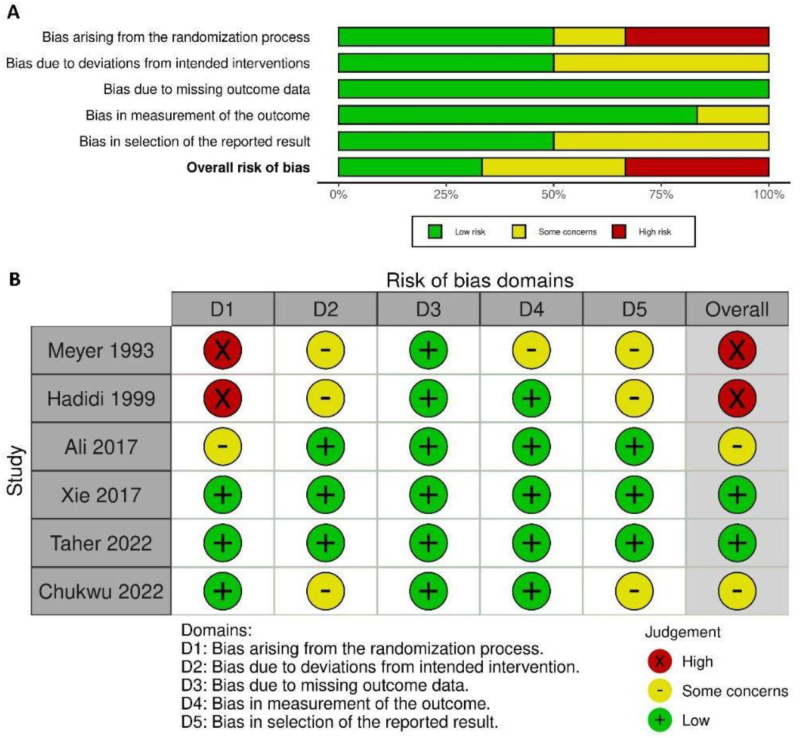
Summary of the risk of bias assessment for the six included randomized controlled trials: green, low risk of bias; red, high risk of bias; or yellow, unknown risk of bias.

Using the Cochrane ROB2 tool for randomized trials, our assessment of the six RCTs revealed that only two of the studies had a high risk of bias due to bias in the randomization process. The graph and summary of ROB are shown in Figure 2.

The quality assessment of twenty-three cohort studies based on the Newcastle‒Ottawa scale (NOS) is found in Supplemental Digital Content Table S2, available at: http://links.lww.com/MS9/A921. Of them, seventeen studies achieved high quality score (score range: 7–9), five studies had fair quality (score range: 3–6). The remaining one study was assessed using NOS for single-arm studies, as this study was single-arm but presented data of both procedures and it showed a low risk of bias in all domains with a low overall risk of bias.

### Primary and secondary outcome results

#### Reduction success rate

**Figure 3.**
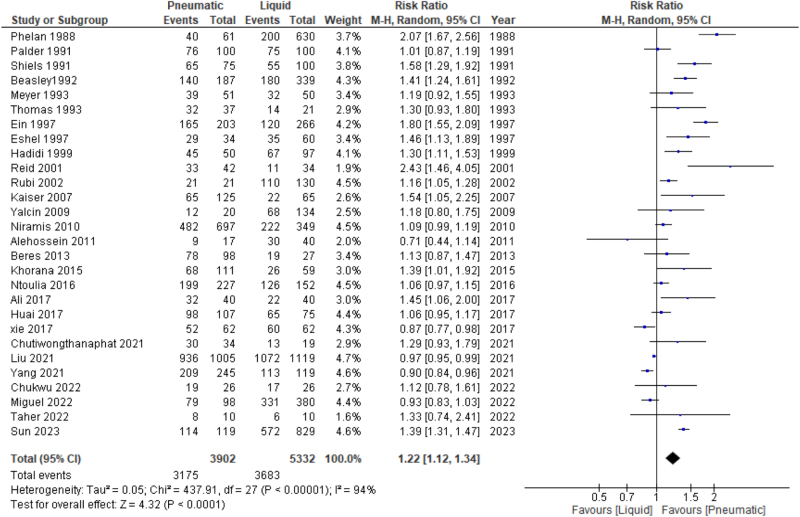
Forest plot for comparison between air and liquid enema reduction regarding the reduction success rate.

A total of 28 studies with 9234 procedures were included in the analysis of this outcome. The meta-analysis demonstrated that pneumatic reduction had a higher success rate than hydrostatic reduction (RR: 1.2 [(95% CI: {1.12–1.34}], *P* < 0.0001), using a random effects model given the high heterogeneity encountered (*I*^2^ = 94%), Figure 3. To resolve heterogeneity, we performed a subgroup analysis based on study design, hospital type, and time of presentation, publication year, country income, and procedure performer. The funnel plot showed publication bias.

##### Subgroup analysis for reduction success rate outcome

Subgroup analysis was performed as follows.

- Study design: The results revealed that within the observational subgroups, pneumatic arms led to a significantly greater reduction (RR: 1.24, 95% CI: [1.12–1.37], *P* < 0.0001), whereas in the RCTs, no significant difference was observed (RR 1.17, 95% CI: [0.93–1.47]; *P* = 0.17). However, significant heterogeneity was noted within both groups (*P* < 0.0001, *I*^2^ = 81%) in the observation group and (*P* < 0.00001, *I*^2^ = 95%) in the RCT subgroup. Sensitivity analysis aimed to address the heterogeneity of the RCT subgroup, revealing significantly superior results in the pneumatic intervention group (RR 1.28, 95% CI: [1.14–1.44]; *P* < 0.0001; *I*^2^ = 0%) (Supplemental Digital Content Figure S1, available at: http://links.lww.com/MS9/A921, Table 2).
- Hospital type: Hospitals were categorized into two groups: children’s hospitals and non-children’s hospitals. Within children’s hospitals, pneumatic reduction demonstrated a higher success rate (RR: 1.24, 95% CI: [1.09–1.42], *P* < 0.0009, *I*^2^ = 93%) compared to non-children’s hospitals (RR: 1.18, 95% CI: [1.00–1.39]; *P* < 0.04; *I*^2^ = 93%). However, significant heterogeneity was observed among studies in both groups (Supplemental Digital Content Figure S2, available at: http://links.lww.com/MS9/A921, Table 2).
- Time of presentation: We classified the duration of the presentation into three groups: before 30, 30–40, and above 40 hours. All groups exhibited heterogeneity, with no discernible differences between the interventions. To address the heterogeneity, sensitivity analysis was conducted, revealing a significant preference for pneumatic reduction (RR: 1.32, 95% CI: [1.14–1.53]; *P* = 0.0002; *I*^2^ = 0%) within the 30–40-hour group. Sensitivity analysis in the remaining two subgroups (<30, >40 h) did not resolve the heterogeneity, and the results showed no significant difference in success rate between the two arms (Supplemental Digital Content Figure S3, available at: http://links.lww.com/MS9/A921, Table 2).
- Publication year: We analyzed the data based on three time periods: before 2000, 2001–2010, and after 2010. Pneumatic reduction had higher success rate than liquid reduction in the subgroup before 2000 (RR: 1.34, 95% CI: [1.23–1.66]; *P* < 0.00001) and the subgroup between 2001 and 2010 (RR: 1.32, 95% CI: [1.06–1.45], *P* = 0.007). However, no significant difference was observed between the pneumatic and liquid methods in the group after 2010. Despite significant heterogeneity across all groups, we were able to address the heterogeneity in the group between 2001 and 2010 using a sensitivity analysis, which revealed favorable results for pneumatics (RR: 1.14, 95% CI: [1.05–1.23], *P* = 0.001, *I*^2^ = 14%) (Supplemental Digital Content Figure S4, available at: http://links.lww.com/MS9/A921, Table 2).
- Country income: According to the World Bank classification of countries’ economies, we analyzed two subgroups: high-income countries and middle- to low-income countries. Pneumatic reduction demonstrated significant superiority in both subgroups; however, the high-income subgroup exhibited better results for pneumatic intervention (RR: 1.35, 95% CI: [1.16–1.56]; *P* < 0.0001) than the low-middle subgroup (RR: 1.12, 95% CI: [1.00–1.24]; *P* = 0.04). There was significant heterogeneity in both groups (*P* < 0.00001, *I*^2^ = 91%) in the high- and in the low – middle-income subgroups (*P* < 0.00001, *I*^2^ = 92 %) (Supplemental Digital Content Figure S5, available at: http://links.lww.com/MS9/A921, Table 2).
- Procedure performer: Eight studies reported on the procedure performer, either a general radiologist or a pediatric radiologist. Among the general radiologists, there were significant differences in the success rate in favor of the pneumatic arm (RR: 1.25, 95% CI: [1.02–1.54]; *P* = 0.04), although significant heterogeneity was present (*P* < 0.05, *I*^2^ = 56%). However, in the pediatric radiologist subgroup, there was no significant difference in the success rate between the two interventions (RR: 1.02, 95% CI: [0.80–1.31]; *P* = 0.86) (Supplemental Digital Content Figure S6, available at: http://links.lww.com/MS9/A921).

**Table 2:** Summary of our analysis results

Analysis		RR and 95% CI	*P*-value	Heterogeneity		N of studies	Conclusion
				*P*-value	*I*^2^		
Reduction success rate		1.2, 95% CI: [1.12, 1.34]	*P* < 0.0001	*P* < 0.00001	*I*^2^ = 94%	28	Higher with pneumatic reduction compared to liquid reduction
Reduction success rate according to publication year	Before 2000s	1.43, 95% CI: [1.23, 1.66]	*P* < 0.00001	*P* < 0.00001	*I*^2^ = 82%	9	Higher with pneumatic reduction compared to liquid reduction
	2001–2010	1.14, 95% CI: [1.05–1.23]	*P* = 0.001	*P* = 0.32	*I*^2^ = 14%	5	Higher with pneumatic reduction compared to liquid reduction
	2011–2023	1.07, 95% CI: [0.97, 1.18]	*P* = 0.2	*P* < 0.00001	*I*^2^ = 92%	14	No significant difference between pneumatic reduction and liquid reduction
Reduction success rate according to study design	RCTs	1.28, 95% CI: [1.14–1.44]	*P* < 0.0001	*P* = 0.83	*I*^2^ = 0%	6	Higher with pneumatic reduction compared to liquid reduction
	Observational studies	1.24, 95% CI: [1.12, 1.37]	*P* < 0.0001	*P* < 0.00001	*I*^2^ = 95%	22	Higher with pneumatic reduction compared to liquid reduction
Reduction success rate according to hospital type	Children’s hospital	1.24, 95% CI: [1.09, 1.42]	*P* < 0.0009	*P* < 0.00001	*I*^2^ = 93%	16	Higher with pneumatic reduction compared to liquid reduction
	Non-children’s hospital	1.18, 95% CI: [1.00, 1.39]	*P* < 0.04	*P* < 0.00001	*I*^2^ = 93%	10	Higher with pneumatic reduction compared to liquid reduction
Reduction success rate according to time of presentation	Before 30 hours	1.11, 95% CI: [0.81, 1.51]	*P* = 0.51	*P* < 0.00001	*I*^2^ = 96%	4	No significant difference between pneumatic reduction and liquid reduction
	30–40 hours	1.32, 95% CI: [1.14–1.53]	*P* = 0.0002	*P* = 0.68	*I*^2^ = 0%	3	Higher with pneumatic reduction compared to liquid reduction
	Above 40 hours	1.08, 95% CI: [0.89, 1.33]	*P* = 0.44	*P* = 0.28	*I*^2^ = 22%	4	No significant difference between pneumatic reduction and liquid reduction
Reduction success rate according to countries income	High-income countries	1.35, 95% CI: [1.16, 1.56]	*P* < 0.0001	*P* < 0.00001	*I*^2^ = 93%	13	Higher with pneumatic reduction compared to liquid reduction
	Middle-low-income countries	1.12, 95% CI: [1.00, 1.24]	*P* = 0.04	*P* < 0.00001	*I*^2^ = 92%	15	Higher with pneumatic reduction compared to liquid reduction
Reduction success rate according to procedure performer	General radiologist	1.25, 95% CI: [1.02, 1.54]	*P* = 0.04	*P* < 0.05	*I*^2^ = 56%	6	Higher with pneumatic reduction compared to liquid reduction
	Pediatric radiologist	1.02, 95% CI: [0.80, 1.31]	*P* = 0.86	*P* = 0.07	*I*^2^ = 70%	2	No significant difference between pneumatic reduction and liquid reduction
Reduction success rate in barium vs. saline		0.91, 95% CI: [0.82, 1.01]	*P* = 0.07	*P* = 0.47	*I*^2^ = 0%	3	No significant differences between barium and saline enema reduction
Perforation rate		0.93, 95% CI: [0.50, 1.71]	*P* = 0.81	*P* = 0.89	*I*^2^ = 0%	14	No significant difference between pneumatic reduction and liquid reduction
Perforation rate in Barium vs. saline		2.05, 95% CI: [0.48, 8.75]	*P* = 0.33	*P* = 0.43	*I*^2^ = 0%	2	No significant differences between barium and saline enema reduction
Recurrence rate		1.07, 95% CI: [0.89, 1.28]	*P* = 0.48	*P* = 0.53	*I*^2^ = 0%	16	No significant difference between pneumatic reduction and liquid reduction
Reduction time		MD = −2.64, 95% CI: [−4.73, −0.55]	*P* = 0.01	*P* = 0.00001	*I*^2^ = 99%	5	Pneumatic group had shorter reduction time compared to the liquid group
Reduction time according to hospital type	Children’s hospital	MD = −4.70, 95% CI: [−6.53, −3.06]	*P* = 0.00001	*P* = 0.89	*I*^2^ = 0%	2	Pneumatic group had shorter reduction time compared to the liquid group
	Non-children’s hospital	MD = −4.43, 95% CI: [−11.80, 2.94]	*P* < 0.24	*P* = 0.12	*I*^2^ = 58%	3	No significant difference between pneumatic reduction and liquid reduction
Length of hospital stay (LOHS)		MD = −1.41, 95% CI: [−5.61, 2.79]	*P* = 0.51	*P* = 0.11	*I*^2^ = 61%	3	No significant difference between pneumatic reduction and liquid reduction
Reduction pressure		MD = −1.52, 95% CI: [−6.65, 3.51]	*P* = 0.55	*P* = 0.07	*I*^2^ = 71%	2	No significant difference between pneumatic reduction and liquid reduction
Fluoroscopy time		MD = −0.13, 95% CI: [−0.95, 0.68]	*P* = 0.75	*P* = 0.57	*I*^2^ = 0%	2	No significant difference between pneumatic reduction and liquid reduction

##### Meta-regression

After adjusting for age and presentation time, a meta-regression analysis was conducted. However, no significant associations were found between these factors and effect size. Supplemental Digital Content Figure S7, available at: http://links.lww.com/MS9/A921, shows a forest plot for the meta-regression.

#### Perforation rate

**Figure 4.**
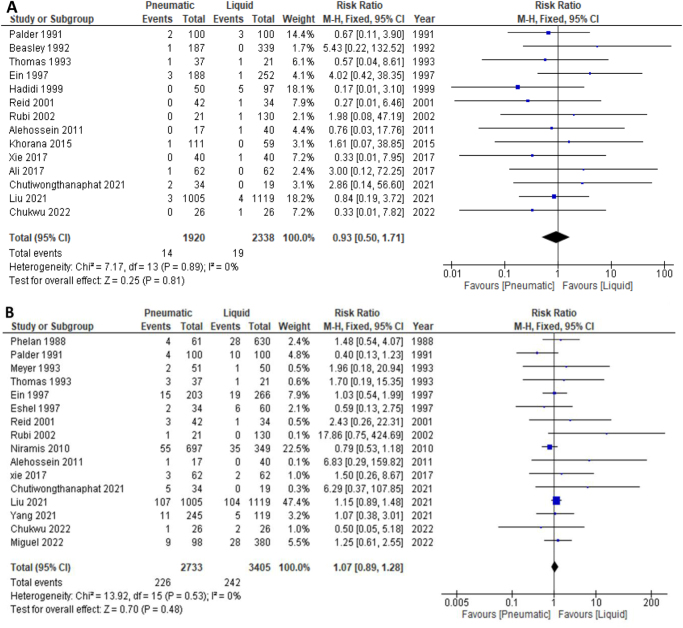
Forest plot for comparison between air and liquid enema reduction regarding (a) perforation rate, (b) recurrence rate.

Fourteen studies were included in the primary analysis of the perforation rate. A total of 1920 patients participated in the pneumatic intervention group, while 2338 participated in the liquid intervention group. The results showed no significant difference in the perforation rate between both techniques (RR: 0.93, 95% CI: [0.50–1.71]; *P* = 0.81; *I*^2^ = 0%), as shown in Figure 4a.

#### Recurrence rate

Sixteen studies were included in the analysis of the recurrence rate. The pneumatic group comprised 2733 patients, while the liquid group had 3405 participants. The pneumatic group had 8.26% recurrence rate, while the hydrostatic group had 7.1%. However, the analysis showed that there was no statistically significant difference in the recurrence rate between pneumatic and liquid interventions (RR: 1.07, 95% CI: [0.89–1.28]; *P* = 0.48; *I*^2^ = 0%), as shown in Figure 4b.

#### Reduction time

**Figure 5.**
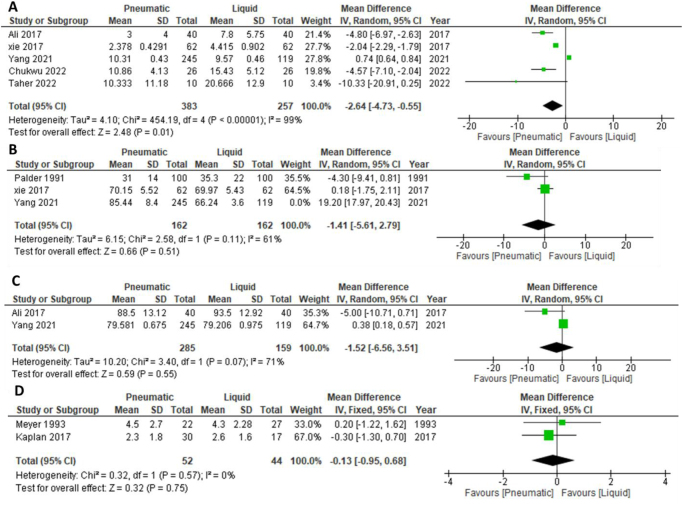
Forest plot for comparison between air and liquid enema reduction regarding (a) reduction time, (b) length of hospital stay, (c) reduction pressure, (d) fluoroscopy time.

This meta-analysis of reduction time comprised five studies with a total of 640 patients. The findings indicated a significantly shorter reduction time in the pneumatic intervention group compared to the liquid intervention group (MD: −2.64, 95% CI: [−4.73 to −0.55], *P* = 0.01, *I*^2^ = 99%), with significant heterogeneity among the studies (*I*^2^ = 99%, *P* = 0.00001), see Figure 5a. Heterogeneity persisted, and it could not be resolved through the leave-one-out test.

Subgroup analysis according to hospital type for reduction time: We conducted subgroup analysis based on hospital type, distinguishing between children’s hospitals and non-children’s hospitals. In children’s hospitals, pneumatic reduction was associated with significantly lower reduction time (MD: −4.70, 95% CI: [−6.53, −3.06], *P* = 0.00001, *I*^2^ = 0%). However, no significant difference between the two interventions was observed in non-children’s hospitals (MD: −1.18, 95% CI: [−3.84, −1.47], *P* = 0.38, *I*^2^ = 100%). Significant heterogeneity was observed among studies in the non-children’s hospital group. A sensitivity analysis was conducted to address the heterogeneity within the non-children’s hospital subgroup, revealing that no significant difference was detected in the pneumatic intervention group (MD: −4.43, 95% CI: [−11.80 to 2.94], *P* < 0.24, *I*^2^ = 58%) (Supplemental Digital Content Figure S8, available at: http://links.lww.com/MS9/A921, and Table 2).

#### Length of hospital stay

The meta-analysis on the LOHS, based on data from three studies involving a total of 688 patients, indicated no significant difference between the pneumatic and liquid intervention groups (MD: 5.14, 95% CI: [−10.27, −20.54]; *P* = 0.51; *I*^2^ = 99%), with considerable heterogeneity (*I*^2^ = 99%, *P* = 0.00001), as shown in Figure 5b.

#### Reduction pressure

The meta-analysis of reduction pressure, including data from two studies and a total of 444 patients, found no significant difference between the pneumatic and liquid intervention groups (MD: −1.52, 95% CI: [−6.56, 3.51]; *P* = 0.55), despite considerable heterogeneity (*I*^2^ = 71%, *P* = 0.07). Consequently, we employed a random effects model to address this heterogeneity, as shown in Figure 5c. Due to the limited number of studies (two studies), a leave-one-out test could not be performed.

#### Fluoroscopy time

Two studies, with a total of 96 patients, were included in the analysis of fluoroscopy time. The results revealed that there was no significant difference between pneumatic and liquid interventions (MD: −0.13, 95% CI: [−0.95, 0.68], *P* = 0.75, *I*^2^ = 0%), see Figure 5d.

#### Mortality

Six independent studies encompassing a total of 2124 patients investigated mortality rates. Among these patients, 534 and 1590 were assigned to the pneumatic and hydrostatic treatment groups, respectively. Notably, two deaths were reported among patients in the hydrostatic group, whereas no fatalities were observed in the pneumatic group.

#### Barium versus saline

No significant differences were found between barium and saline in terms of success rate (RR: 0.91, 95% CI: [0.82–1.01]; *P* = 0.07) and perforation rate (RR: 2.05, 95% CI: [0.48–8.75]; *P* = 0.33), and there was no heterogeneity (Supplemental Digital Content Figure S9, available at: http://links.lww.com/MS9/A921).

## Discussion

The management of pediatric intussusception has evolved dramatically, transitioning from surgical interventions to nonoperative treatments. Currently, surgery is reserved for patients who are unstable, exhibit signs of peritonitis or perforation, or when enema reduction fails[42]. Enhancing nonsurgical management aims to achieve safe, simple, and early reduction via contrast enema while minimizing the risks of radiation exposure and peritoneal contamination from iatrogenic perforation[38]. Two main non-operative methods are widely used: liquid reduction and air reduction. The former involves the use of barium, iodinated contrast material, water, or saline solution, which can lead to electrolyte imbalance. The latter employs air, CO_2_, or oxygen administered via a rectal catheter at a mean pressure of 80–120 mmHg and is believed to have a higher success rate with fewer complications[10]. Therefore, our study aimed to shed light on this debate by evaluating the efficacy and safety of air versus liquid enema reduction in the treatment of pediatric intussusception.

We included 29 studies in our meta-analysis. Our findings revealed a significantly higher success rate in the pneumatic group (81.3%) compared to the liquid reduction group (69.1%). Moreover, the pneumatic group exhibited a lower perforation rate and a shorter reduction time. Importantly, there were no significant differences between the two interventions in terms of hospital stay duration, reduction pressure, and fluoroscopy time. These results underscore the potential advantages of pneumatic reduction in the nonoperative management of pediatric intussusception, highlighting its efficacy and safety. The superiority of pneumatic reduction may be attributed to several biomechanical and procedural factors. Air has lower viscosity than liquid, allowing for rapid and uniform colon insufflation, which reduces resistance during the reduction process. The transparent air column during fluoroscopy also provides better real-time visualization, enhancing procedural accuracy. Additionally, the compressibility of air allows for more controlled pressure adjustments, reducing the risk of abrupt distension and perforation. These factors likely contribute to its higher success and efficiency compared to hydrostatic methods.

Our current results align with two previous studies, demonstrating an improved success rate with air enema. Sadigh *et al*[10] compared the success rates of pneumatic and liquid reduction methods in single-arm studies, revealing higher success rates for pneumatic methods (82.7%) compared to liquid reduction (69.6%)[41]. Another meta-analysis by Beres *et al*[9] also found that pneumatic reduction had a lower failure rate. See Table 3 for more comparison of our study with these two studies.

**Table 3:** Comparison of our meta-analysis with another published meta-analysis

	Beres 2013[9]	Gray 2014[43]	Sadigh 2015[10]	Our study
Total studies included in MA	19	69	101	29
Type of studies included	Comparative studies	Single arm studies	Single arm studies	Comparative studies
Total sample size	5549	15 375	32 451	9281
Language of included studies	English, Spanish	English	English	English
Number of outcomes assessed	2	1	3	9
Subgroup analysis based on	Study design	Study quality, year of study publication, and country of origin	Enema reduction guidance, publication period, country of publication, recruitment	Study design, presentation time, enema reduction guidance, publication period, country of publication (income), procedure performer
Meta regression covariates	NR	Age	NR	Age, presentation time
Reduction failure rate	Lower with pneumatic	NR	NR	NR
Reduction success rate	NR	NR	Higher with pneumatic	Higher with pneumatic
Perforation rate	No significant difference	NR	No significant difference	Lower with pneumatic
Recurrence rate	NR	Higher recurrence in contrast- enema and air enema than non-contrast enema	No significant difference	No significant difference
Length of hospital stay	NR	NR	NR	No significant difference
Reduction time	NR	NR	NR	Favor pneumatic
Reduction pressure	NR	NR	NR	No significant difference
Fluoroscopy time	NR	NR	NR	No significant difference
Barium vs. saline	NR		NR	No significant difference in success rate and perforation rate
Mortality	NR	NR	NR	2 deaths in liquid group, none in pneumatic

Our Subgroup analysis for success rate outcome revealed that pneumatic reduction was superior in both the cohort and randomized control trials. Additionally, subgroup analysis of studies comparing hydrostatic and pneumatic techniques for treating intussusception initially favored pneumatic methods in studies published before 2011. However, advancements in this field have led to a shift, with no significant difference observed between the two techniques from 2011 to 2023. This shift underscores the dynamic evolution and enhancement of noninvasive techniques, particularly pneumatic methods, in pediatric interventions, such as intussusception treatment. The convergence of success rates between hydrostatic and pneumatic reduction over time highlights the pivotal role of ongoing research and technological advancements in refining medical practices and improving patient outcomes.

This progress is crucial for advancing healthcare standards and treatment efficacy in clinical settings. Operator expertise influenced success rates. Pneumatic reduction showed higher success when performed by general radiologists, while no difference was observed between techniques when conducted by pediatric radiologists. This suggests that pediatric radiologists are proficient in both methods, while non-specialists may find pneumatic reduction technically easier or more forgiving.

Further subgroup analysis based on presentation time indicated no significant difference between the two interventions after 40 hours, suggesting that both pneumatic and liquid reduction techniques are feasible within this timeframe. However, conflicting reports exist regarding the impact of delayed presentation on outcomes. Some studies suggest that delayed diagnosis increases the risk of bowel complications and reduces the success rate of enema reduction[44]. While others argue against this association^[45,46]^. These discrepancies may arise from differences in statistical analysis methodologies, particularly in adjusting for multiple factors. In our meta-regression analysis, which evaluated the mean time of presentation across studies, we found that presentation timing did not significantly affect the success rate outcome. This indicates that the efficacy of both pneumatic and liquid reduction methods remains consistent across varying presentation times considered in the studies.

After successful enema reduction, recurrence of intussusception is a recognized complication, occurring in 9%–15% of patients within a few days[10]. The higher recurrence with air reduction may reflect earlier discharge policies and shorter observation periods following successful pneumatic reduction, potentially allowing some cases of early recurrence to go undetected or occur post-discharge. Another possibility is that air insufflation may lead to a less complete reduction in borderline cases, although this remains speculative[47]. Our study found that pneumatic reduction had an 8.26% recurrence rate, while hydrostatic reduction had 7.1%, with no significant differences. A similar result was found by Renwick *et al*[48] who observed a recurrence rate of 7.9% with gas enema reduction, like the 8.9% recurrence rate with barium enema. However, Gray *et al*[43] reported a higher recurrence rate in contrast enema reduction (12.7%) than air (8.5%) and non-contrast enema reduction (7.5%).

Another potential complication is intestinal perforation, although rare, which can result from the application of high enema pressures[49]. During air enema procedures, high intestinal lumen pressure can cause significant expansion of the intestinal tube, and excessive or abrupt pressure may lead to a tense pneumoperitoneum, potentially causing intestinal perforation[50]. In liquid enema reduction, perforation can result in intra-abdominal fecal contamination, which, if not promptly treated, can lead to severe complications and jeopardize patient safety. Our meta-analysis results indicate no significant difference in the perforation rates between the two techniques, which aligns with earlier findings by Sadigh *et al*[10] and Beres *et al*[9]. Additionally, mortality was reported in two patients in the hydrostatic enema group out of 5349 patients (0.037%), whereas no deaths were reported in the pneumatic group.

Regarding reduction pressure outcome, other studies have shown that air enema pressure is greater than liquid enema pressure, leading to a greater success rate^[51,52]^. However, our meta-analysis included only two studies, and results did not reveal a significant difference in the reduction of pressure. Notably, heterogeneity was observed, likely due to methodological variations – for instance, Yang *et al* used a novel device developed locally for enema delivery, possibly influencing pressure readings.

Our meta-analysis showed that the reduction time was significantly shorter in the pneumatic group, which is consistent with the findings of several studies^[17,23,24,53]^. The shorter reduction time with pneumatic reduction is attributed to the low viscosity of air, which allows for rapid colon filling, making the process quicker and more effective than liquid enema. Ali *et al*[23] reported that intussusception experiences less friction during pneumatic reduction due to air insufflation under pressure, facilitating an easier reduction process. In terms of hospitalization length, no differences were observed between the two procedures because both were nonsurgical and did not require extended hospital stays.

### Strengths and limitations

The primary strength of this study is its inclusion of 29 comparative studies involving both liquid and pneumatic enema treatments in a total of 9281 children. By excluding single-arm trials, we significantly enhanced the strength and reliability of the evidence. This approach allowed us to draw more accurate conclusions and provide better recommendations. Additionally, our study employed a comprehensive search strategy, meticulous data extraction, and quality assessment conducted by two independent reviewers, along with robust statistical analysis. We conducted subgroup analyses based on study design, hospital type (children vs. non-children), presentation time, and publication year. Further subgroup analyses were performed for the countries of the included studies and the enema type (barium vs. liquid). We also conducted a meta-regression analysis for age and presentation time.

However, our study has several limitations. Most of the included studies had cohort designs, and not all outcomes, such as radiation dosage and fluoroscopy time, were evaluated in the included studies. Future comparative RCTs examining these outcomes are recommended. Additionally, more research is needed to compare the effectiveness and success rate of enema reduction between pediatric surgeons and radiologists. And studies examining the success rate according to different presentation time, age, and gender. Furthermore, we limited our search to publications in English, which may have excluded relevant studies published in other languages.

## Conclusion

Intussusception is a common abdominal emergency in infants and children, where early reduction is crucial to prevent complications and improve patient outcomes. Pneumatic reduction has been found to be more successful and quicker than hydrostatic reduction. However, both techniques show no significant differences in perforation rate, recurrence rate, length of hospital stay, reduction pressure, and fluoroscopy time. Further comparative studies are recommended to examine radiation dose and fluoroscopy time.

## Data Availability

All data generated or analyzed during this study are included in this published article or in the data repositories listed in the references.

## Research registration unique identifying number (UIN)

PROSPERO, CRD42024504420.
